# Comparison of microwave ablation treatments in patients with renal secondary and primary hyperparathyroidism

**DOI:** 10.1080/0886022X.2019.1707097

**Published:** 2020-01-12

**Authors:** Haoyang Ma, Chun Ouyang, Yaoyu Huang, Changying Xing, Chen Cheng, Wei Liu, Donglan Yuan, Ming Zeng, Xiangbao Yu, Haibin Ren, Yanggang Yuan, Lina Zhang, Fangyan Xu, Ying Cui, Wenkai Ren, Hui Huang, Hanyang Qian, Boqiang Fan, Ningning Wang

**Affiliations:** a Department of Nephrology, the First Affiliated Hospital of Nanjing Medical University, Jiangsu Province Hospital, Nanjing, China; b Department of Oncology, the First Affiliated Hospital of Nanjing Medical University, Jiangsu Province Hospital, Nanjing, China; c Department of Nuclear Medicine, the First Affiliated Hospital of Nanjing Medical University, Jiangsu Province Hospital, Nanjing, China; d Department of Nephrology, Henan Key Laboratory for Kidney Disease and Immunology, Henan Provincial People’s Hospital, Zhengzhou University People’s Hospital, Henan, China; e Department of Biostatistics, School of Public Health, Nanjing Medical University, Nanjing, China

**Keywords:** Secondary hyperparathyroidism, chronic kidney disease-mineral and bone disorder, primary hyperparathyroidism, microwave ablation, safety, efficacy

## Abstract

**Purpose:**

Microwave ablation (MWA) is feasible for severe renal secondary hyperparathyroidism (SHPT) and primary hyperparathyroidism (PHPT) patients ineligible for parathyroidectomy (PTX). Here we compared the clinical manifestations and characteristics of parathyroid glands in these two groups, and summarized the techniques, safety and efficacy of MWA.

**Methods:**

Baseline clinical characteristics, ablation-related techniques, adverse events/complications, and efficacy were recorded.

**Results:**

In SHPT group, malnutrition, cardiovascular/pulmonary complications, and abnormal bone metabolism were severe. SHPT patients had more hyperplastic parathyroid glands. The volume of each gland was smaller, and the time of ablation for a single parathyroid was shorter in the SHPT group, although there were no significant differences compared with patients in the PHPT group. Three patients in both groups had recurrent laryngeal nerve injuries and all recovered, except for one SHPT patient. By the end of follow-up, serum iPTH levels had decreased from 2400.26 ± 844.26 pg/mL to 429.39 ± 407.93 pg/mL (*p* < .01) in SHPT and from 297.73 ± 295.32 pg/mL to 72.22 ± 36.51 pg/mL in PHPT group (*p* < .01). Hypocalcemia was more common (*p* < .001) and serum iPTH levels were prone to rebound in SHPT patients after MWA.

**Conclusion:**

MWA can be reserved for those who had high surgical risks because of less invasiveness. Injuries of recurrent laryngeal nerves should be noticed. The health status, perioperative, and intraoperative procedures were more complicated and all parathyroids found by ultrasound should be ablated completely in SHPT patients.

## Introduction

Secondary hyperparathyroidism (SHPT) is an important aspect of chronic kidney disease-mineral and bone disorder (CKD-MBD), with hyperplastic parathyroid glands and high levels of serum intact parathyroid hormone (iPTH) [1]. The clinical manifestations include ostealgia, bone deformity, pathologic fractures, ectopic calcifications in soft tissues, arrhythmia, heart failure, cognitive impairments, and other abnormalities [2,3]. Current treatments for SHPT include dietary phosphorus restriction, modification of dialysis prescriptions, phosphate binders, calcitriol or vitamin D analogs, and calcimimetics [4]. The National Kidney Foundation’s Kidney Disease Outcomes Quality Initiative (KDOQI) recommends that parathyroidectomy (PTX) should be taken into account when pharmacologic therapy has failed either because of drug resistance or side effects [5]. However, SHPT patients with serious cardiac and/or pulmonary comorbidities are at increased risk of complications of general anesthesia. To alleviate clinical symptoms, thermal ablation can be introduced as an alternative treatment [6–8].

Primary hyperparathyroidism (PHPT) is the most common cause of hypercalcemia. The autonomous hypersecretion of PTH may be caused by parathyroid adenoma, hyperplasia, or carcinoma [9]. Solitary parathyroid adenomas account for approximately 80% of cases of PHPT [10]. It predominantly affects elderly populations and women 2–3 times as often as men [11]. Clinical manifestations include recurrent urinary stones, skeletal, gastrointestinal, or cardiovascular disorders [12]. PTX is suggested in all cases of symptomatic PHPT.

In recent years, percutaneous ablation techniques have been developed for use in patients with renal SHPT and PHPT [13]. However, the pathogenesis of PHPT and SHPT are different and there has been no research comparing thermal ablation treatment in these two groups. Here we compared the clinical manifestations, characteristics of parathyroids, and evaluated the safety and efficacy of MWA of these two groups of patients. The aim of this study was to propose the key operating points of MWA and improve the treatment outcomes of these two groups of patients.

## Materials and methods

### Patients

This was a retrospective study of patients with SHPT (*n* = 16) and PHPT (*n* = 17) who underwent MWA treatment. The SHPT patients were hospitalized from November 2014 to November 2016, and the PHPT patients were hospitalized from January 2015 to November 2016. The endpoint of follow-up was 30 June 2018. All the patients in the study were informed of the potential dangers of the ablative technique, and signed the informed consent before they underwent MWA treatments. The study protocols were approved by the Research Ethics Committee of the First Affiliated Hospital of Nanjing Medical University, People’s Republic of China (The approved number 2015-SR-143). All of the procedures performed in studies were in accordance with the 1964 Helsinki declaration and its later amendments or comparable ethical standards.

### Inclusion and exclusion criteria for SHPT and PHPT patients

**Figure 1.**
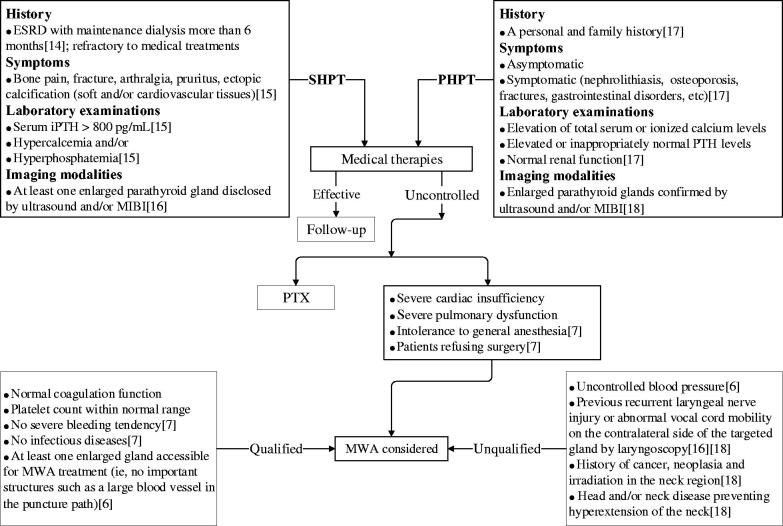
A flowchart of diagnostic procedures and inclusion and exclusion criteria for patients with SHPT and PHPT undergoing MWA. SHPT: secondary hyperparathyroidism; PHPT: primary hyperparathyroidism; MWA: microwave ablation; ESRD: end stage renal disease; iPTH: intact parathyroid hormone; MIBI: ^99m^Tc sestamibi scintigraphy; PTX: parathyroidectomy.

Inclusion and exclusion criteria for SHPT and PHPT patients are listed in Figure 1.

**Inclusion criteria:** (1) SHPT patients refractory to medical treatments, such as restricting the intake of phosphate, using phosphate binders, active vitamin D, calcium-sensing receptor agonists, etc; (2) patients ineligible for PTX because of intolerance to general anesthesia (severe cardiac/pulmonary dysfunctions) or refused surgery; (3) coagulation function and platelet count within normal range; no severe bleeding and infectious diseases; (4) at least one enlarged gland accessible for MWA treatment (i.e., no important structures such as a large blood vessel in the puncture path).

**Exclusion criteria:** (1) patients with uncontrolled blood pressure prior to MWA; (2) patients with previous recurrent laryngeal nerve injuries or abnormal vocal cord mobility on the contralateral side of the targeted gland confirmed by laryngoscopy; (3) known history of parathyroid cancer or other neoplasia in the neck region; history of neck irradiation; head and/or neck disease-preventing hyperextension of the neck.

### Examinations and preparations prior to MWA

#### Laboratory examinations

Routine blood tests were performed using an LH-750 Hematology Analyzer (Beckman Coulter, Inc., Fullerton, CA, USA). Routine coagulation parameters were measured with a coagulation analyzer (CS5100; Sysmex Corporation, Belgium). Biochemical indices were measured with an automatic biochemical analyzer (AU5400; Olympus Corporation, Japan). Serum iPTH levels were measured using a UniCel DxI800 Access Immunoassay System (Beckman Coulter, Inc., Fullerton, CA, USA).

#### Cardiac and pulmonary function assessments

These assessments included a routine electrocardiogram (ECG), chest X-ray, two-dimensional echocardiography, and pulmonary function testing.

#### Imaging examinations of parathyroid glands

**Figure 2.**
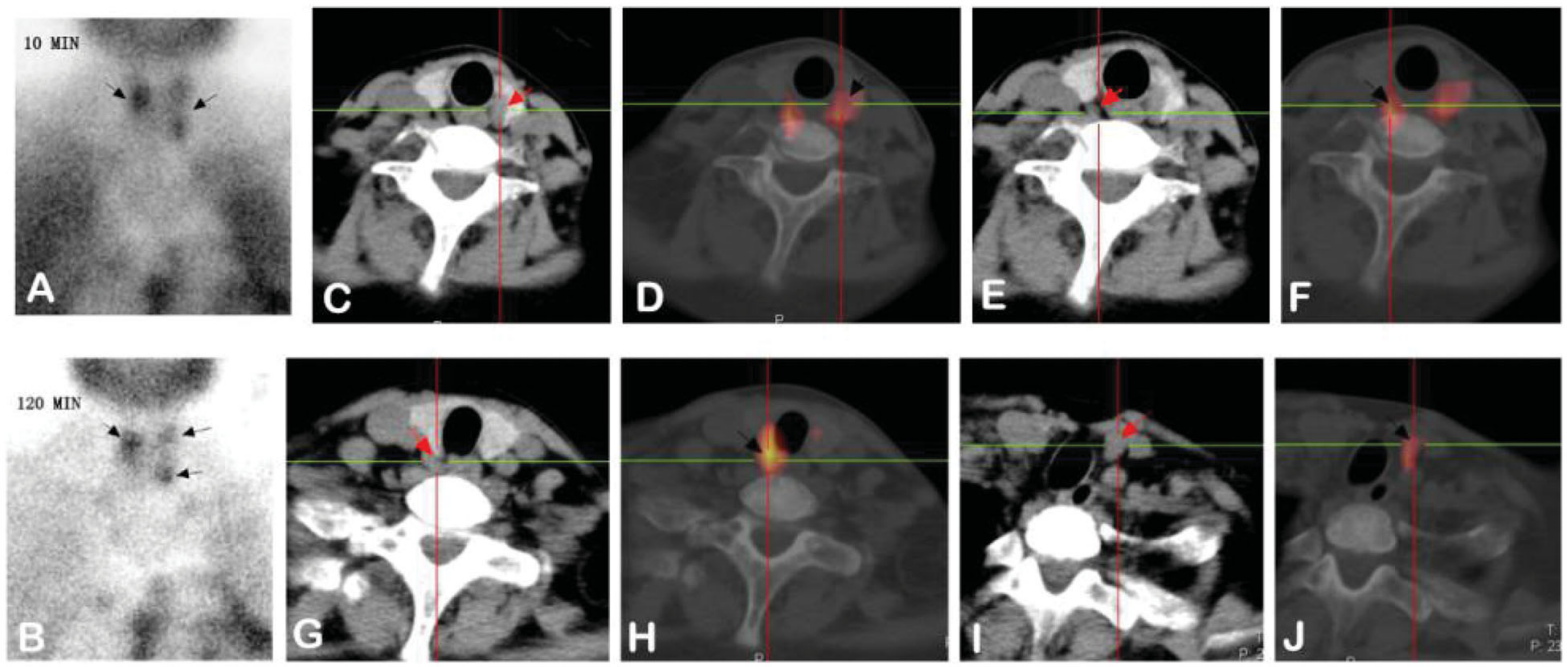
Images from a 28-year-old female diagnosed with SHPT. (A,B): Dual phase 99mTc-MIBI planar scintigraphy of parathyroid glands. (A) early phase (after 10 minutes post injection of tracer) – showing radioactivity uptake in thyroid with increased distribution in left lobe and upper pole of right lobe. (B) late phase (after 120 min) – activity of wash-out from thyroid and retention in hyperfunctioning parathyroid glands (upper pole and inferior pole of left thyroid, upper pole of right thyroid, black arrows). (C–H): CT alone (C/E/G) and 99mTc-MIBI SPECT/CT images (D/F/H) of the same cross-section views showing the right upper (C and D), the left upper (E and F) and inferior (G and H) hyperfunctioning parathyroid glands (arrows). (I,J): CT alone (I) and 99mTc-MIBI SPECT/CT images (J) of the same cross-section views showing a new hyperfunctioning parathyroid gland that was not found in 99mTc-MIBI planar imaging in the inferior pole of right thyroid (arrows).

**Figure 3.**
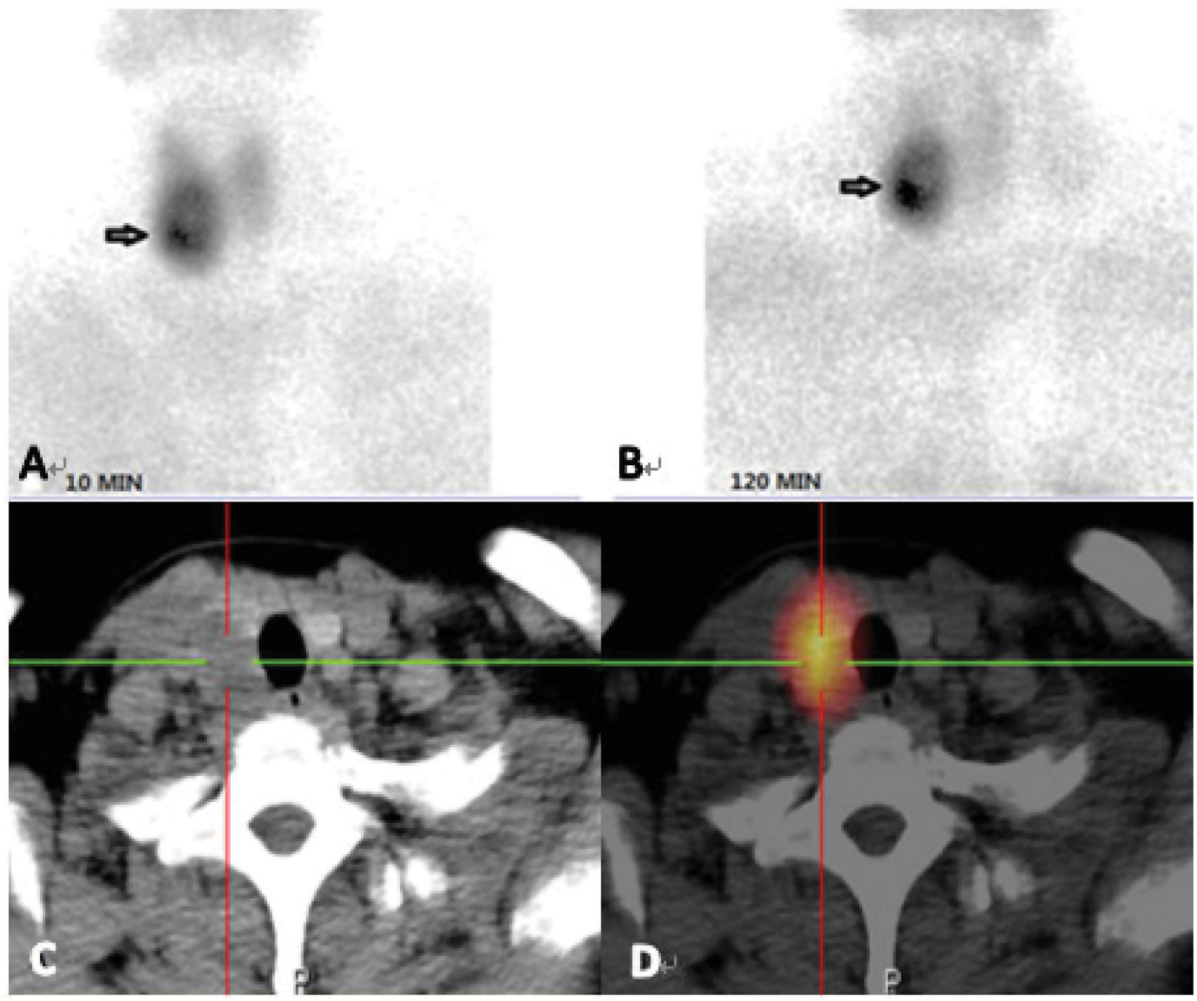
Images from a 41-year-old female diagnosed with PHPT. Her serum levels of iPTH and calcium on admission were 726.80 pg/mL and 2.97 mmol/L, respectively. (A, B): Dual phase ^99m^Tc-MIBI planar scintigraphy of the parathyroid glands, (A) 10min, (B) 120 min. CT alone (C) and SPECT/CT (D) of the same cross-section, showing the right inferior parathyroid. The best diagnostic performance is achieved by combination of a detailed morphological image (CT) and functional, scintigraphic map (SPECT/CT).

These examinations included ^99m^Tc-MIBI-SPECT/CT (Figures 2,3) and high-resolution ultrasonography (USG), which can help detect ectopic parathyroid glands. The size of the nodule, the internal texture (solid or cystic), the shape, the echogenicity, the margin, the presence of calcification, and adjacent structures should be carefully scrutinized by USG [14].

### Key points of MWA techniques

**Figure 4.**
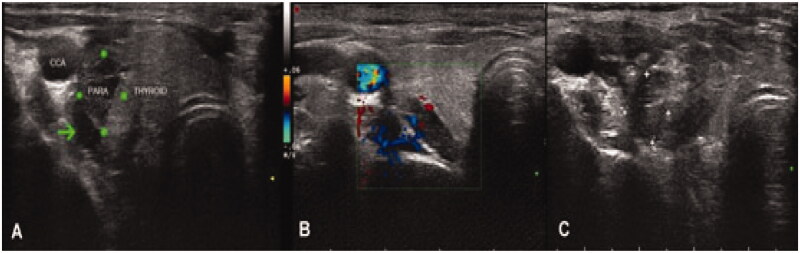
Images from a 29-year-old female diagnosed with severe SHPT. (A) Before MWA, a liquid insulation layer (the arrow head) was created to protect the adjacent recurrent laryngeal nerves and vessels from thermal injury. The size of the parathyroid gland at the superior right position detected by ultrasound was 1.7*1.3*0.6 cm. (B) Color Doppler image showed abundant blood flows in the gland before MWA. (C) After MWA，the local echo of the nodule was not uniform when detected by ultrasound. SHPT : secondary hyperparathyroidism.

MWA was performed by an experienced interventional doctor using a KY2000 ablation system (Nanjing Kangyou Applied Research Institute, Nanjing, China). During the procedure, each patient was accompanied by an anesthesiologist who could provide emergency treatment in the event of dyspnea, arrhythmia, or cardiopulmonary arrest. Key points of MWA techniques include the following: (1) Before MWA, intravenous access was obtained. Under ECG monitoring, the patient was placed in the supine position with the neck region fully exposed and sterilized. Local anesthesia was carried out with 2% lidocaine injected at the puncture site under USG guidance. Before MWA, abundant blood flow in the gland was shown by color Doppler imaging (Online Resource1). (2) A liquid insulation layer was used as a hydrodissection technique. Specifically, a mixture of lidocaine and normal saline was injected into the area around the parathyroids to create a heat insulation layer (Figure 4(A), Online Resource 2) to protect the adjacent recurrent laryngeal nerves and vessels from the thermal injury [15]. (3) Under the guidance of real-time ultrasound (Figure 4(B)), the ablation was started with the power between 25 and 45 W. The ablation duration, times, and intervals of each parathyroid gland were determined according to the characteristics and adjacent structures of each target gland in order to avoid thermal injury (Online Resource3). (4) The ablation was considered complete when the parathyroid gland turned transient hyperechoic and the color flow signals disappeared (Figure 4(C), Online Resource 4). (5) The ablation was continued if the patient did not have hoarseness or dyspnea. Otherwise, it was terminated immediately.

### Statistical analysis

Statistical analysis was performed using SPSS version 22. Continuous variables were presented as mean ± SD or median (interquartile range), and categorical variables were presented as frequencies. The baseline characteristics of SHPT and PHPT patients were compared using the *t*-test (for normal distribution data) or Mann-Whitney U test (for skewed data) for continuous variables. Categorical variables were presented as frequencies and analyzed using chi-squared or Fisher exact test. A two-sided *p*-value <.05 was considered statistically significant unless stated otherwise.

## Results

### Comparisons of baseline demographic and clinical characteristics of PHPT and SHPT patients who received MWA

Baseline demographic and clinical characteristics of PHPT and SHPT patients who received MWA were listed in Table 1. Compared with PHPT patients, the SHPT group had lower levels of body mass index (BMI) and higher proportions of cardiovascular and pulmonary diseases including coronary heart disease and pulmonary hypertension. BMI is an integral part of anthropometric assessments and is a widely used indicator to assess nutritional status [16]. The clinical symptoms of SHPT patients, such as ostealgia, arthralgia, and pruritus, were more obvious (Table 1).

**Table 1.** Baseline clinical characteristics of PHPT and SHPT patients who received MWA.

Variables	PHPT (*n* = 17)	SHPT (*n* = 16)	*p* Value
Demographics			
Age (years)	57.53 ± 15.18	55.38 ± 15.17	.686
Male/Female	2/15	7/9	.057
BMI (kg/m^2^)	23.84 ± 3.00 (*n* = 16)	19.88 ± 2.53	**<.001**
BMI < 23 (kg/m^2^), *n*(%)	7/16 (*n* = 16)	15/16	**.006**
Systolic BP (mmHg)	123.71 ± 11.86	138.06 ± 32.86	.116
Diastolic BP (mmHg)	74.94 ± 6.99	79.31 ± 16.12	.315
Dialysis duration (months)	0	94.88 ± 60.01	**<.001**
Comorbidities, *n*(%)			
Hypertension	4/17	8/16	.157
Diabetes Mellitus	2/17	2/16	1.000
Coronary heart disease	2/17	8/16	**.026**
Previous history of PCI	0/17	2/16	.227
LVEF (%)	NA	50.24 ± 16.83	NA
ST-T changes	6/17	9/12^a^	.060
Cardiothoracic ratio >0.5	1/17	11/14^a^	**<.01**
Pulmonary hypertension	1/17	9/16	**.002**
mild (30–40mmHg)	0/17	4/16	**.044**
moderate (40–70mmHg)	0/17	3/16	.103
severe (>70mmHg)	1/17	2/16	.601
PAP (mmHg)	NA	48.08 ± 23.64 (*n* = 12)	NA
Mild pulmonary dysfunction	NA	6/15^a^	NA
Thrombocytopenia	0/17	2/16	.227
Symptoms and signs on admission, *n*(%)			
Bone fracture	1/17	3/16	.335
Ostealgia	0/17	8/16	**.001**
Arthralgia	0/17	9/16	**<.001**
Pruritus	0/17	7/16	**.003**
Ectopic calcifications in soft tissues	0/17	2/16	.227
Calciphylaxis	0/17	1/16	.485

*Note*: Data are mean ± standard deviation, or numbers and percentages, as appropriate. PHPT: primary hyperparathyroidism; SHPT: secondary hyperparathyroidism; BMI: body mass index; BP: blood pressure; PCI: percutaneous coronary intervention; LVEF: left ventricular ejection fraction; PAP: pulmonary artery pressure; NA: not available.

^a^ Some patients did not undergo the indicated examinations.

Note: Bold Numbers mean statistically significant, *P* < 0.05

SHPT patients had higher levels of serum iPTH (*p* < .001), phosphorus (*p* < .001), and alkaline phosphatase (ALP) (*p* = .001) values compared with PHPT patients. However, there were no significant differences in serum calcium levels (*p* = .086). Furthermore, SHPT patients had much more serious anemia, malnutrition, lipid, and bone metabolism disorders (Table 2). Data from positive parathyroid glands detected by USG and MIBI in PHPT and SHPT patients before MWA were shown in Table 3.

**Table 2.** Laboratory results of PHPT and SHPT patients before MWA.

	PHPT (*n* = 17)	SHPT (*n* = 16)	*p* Value
Laboratory values			
Hemoglobin (g/L)	129.65 ± 15.56	105.19 ± 16.44	**<.001**
Hematocrit (%)	0.40 ± 0.05	0.33 ± 0.06 (*n* = 15)	**.002**
Platelet (*10^9^/L)	214.65 ± 79.81	142.19 ± 50.67	**.004**
Glucose (mmol/L)	5.46 ± 0.85	4.82 ± 1.38	.121
Creatinine (μmol/L)	64.23 ± 30.43	723.81 ± 276.67	**<.001**
BUN (mmol/L)	5.36 ± 2.81	21.83 ± 8.75	**<.001**
Albumin (g/L)	41.48 ± 5.90	37.30 ± 3.69	**.023**
ALT (U/L)	25.09 ± 25.11	12.53 ± 6.37	.061
AST (U/L)	23.99 ± 11.21	16.68 ± 6.41	**.029**
HDL cholesterol (mmol/L)	1.41 ± 0.47	1.01 ± 0.32	**.009**
LDL cholesterol (mmol/L)	3.33 ± 0.82	2.47 ± 0.51	**.001**
TC (mmol/L)	5.24 ± 1.19	3.87 ± 0.58	**<.001**
Triglyceride (mmol/L)	1.51 ± 0.84	1.88 ± 1.82	.453
Bone metabolism panel			
Calcium (mmol/L)	2.72 ± 0.30	2.56 ± 0.19	.086
Phosphorus (mmol/L)	0.96 ± 0.22	1.92 ± 0.42	**<.001**
ALP (u/L)	121.60 ± 64.78	941.54 ± 844.80	**.001**
iPTH (pg/mL)	297.73 ± 295.32	2400.26 ± 844.26	**<.001**

*Note*: Data are mean ± standard deviation. ALT: alanine aminotransferase; AST: aspartate aminotransferase; HDL: high density lipoprotein; LDL: low density lipoprotein.

Note: Bold Numbers mean statistically significant, *P* < 0.05

**Table 3.** Imaging data of parathyroid glands from PHPT and SHPT patients before MWA.

	PHPT (*n* = 17)			SHPT (*n* = 16)		
	USG	MIBI	*p* Value	USG	MIBI	*p* Value
Location of glands, *n* (%)						
Left superior	3/17	2/17	1.000	13/16	13/16	1.000
Left inferior	10/17	9/17	.730	14/16	14/16	1.000
Right superior	2/17	2/17	1.000	12/16	10/16	.704
Right inferior	5/17	3/17	.688	13/16	10/16	.433
Ectopic	0/17	0/17	/	0/16	0/16	/
Number of enlarged glands, *n* (%)						
0	0/17	1/17	1.000	0/16	0/16	/
1	14/17	16/17	0.601	2/16	1/16	1.000
2	3/17	0/17	.227	0/16	4/16	.101
3	0/17	0/17	/	6/16	6/16	1.000
4	0/17	0/17	/	8/16	4/16	.273
Total number of glands	20	16	/	52	47	/

*Note*: Data are percentages and numbers. PHPT: primary hyperparathyroidism; SHPT: secondary hyperparathyroidism; USG: ultrasonography; MIBI: ^99m^Tc sestamibi scintigraphy.

Note: Bold Numbers mean statistically significant, *P* < 0.05

### Comparisons of technical parameters for ablation between SHPT and PHPT patients

The median number of parathyroid glands ablated for each patient in the SHPT and PHPT groups was 3 and 1, respectively (*p* < .001). Compared with SHPT patients, PHPT patients had larger volumes and maximum diameters of single glands and longer ablation times for each gland. However, there were no statistically significant differences. SHPT patients usually underwent MWA treatments at two separate times in order to avoid bilateral recurrent laryngeal nerve injuries, whereas PHPT patients were treated only once (Table 4).

**Table 4.** Technical parameters and complications/side effects of parathyroid MWA in PHPT and SHPT patients.

Variables	PHPT (*n* = 17)	SHPT (*n* = 16)	*p* Value
Median number of ablated glands	1	3	**<.001**
Volume of a gland (mm^3^)	1033.06 (706.50–2519.85)	581.82 (394.90–901.47) (*n* = 12)	.322
Maximum diameter of a gland (mm)	20.00 (13.50–25.5)	17.50 (15.25–19.00) (*n* = 12)	.753
Ablation time for a gland (sec)	107.00 (43.50–254.75)	69.50 (47.31–100.69)	.132
Ablation power (W)	35 (35.00–40.00)	35 (35–35)	**.013**
Cases of treatment in separate times, *n* (%)	0/17	15/16	**<.001**
Cases of repeated ablation of a gland, *n* (%)	2/17	2/16	1.000
Complications/side effects, *n* (%)			
Hemorrhage/hematoma	0/17	0/15^a^	/
Hoarseness	3/17	3/13^a^	1.000
Hypocalcemia	2/17	14/16	**<.001**
Intraoperative pain	5/17	5/13^a^	.705

*Note*: Data are numbers, medians with interquartile range or percentages, as appropriate. PHPT: primary hyperparathyroidism; SHPT: secondary hyperparathyroidism.

^a^ Medical records of the complications/side effects were not described in three patients.

### Safety of MWA

Three SHPT patients had symptoms of recurrent laryngeal nerve injury, manifesting as voice hoarseness without dyspnea. Two of them recovered within 1 week after ablation, and another remained hoarse at the end of follow-up (six months after ablation). Transient hoarseness during MWA was encountered in three PHPT patients, all of whom recovered within 1 day after administration of mecobalamine, dexamethasone and nebulized inhalation therapies. Hypocalcemia occurred in 14 SHPT patients within 4 days and 2 PHPT patients within 1 day after ablation; this was corrected with oral/intravenous supplementation of calcium. None of the patients experienced hemorrhage/hematoma, infection, or skin scalding at the puncture sites (Table 4).

### Efficacy of MWA

#### Changes of serum iPTH and calcium levels in patients with SHPT and PHPT before and after MWA

In SHPT patients who received MWA, serum iPTH levels on baseline, 20 min–24 h after MWA were 2400.26 ± 844.26 pg/mL, 403.60 ± 295.32 pg/mL and 267.31 ± 199.06 pg/mL, respectively (*p* < .001 *vs*. baseline). Serum iPTH levels on discharge days and by the end of follow-up were 423.43 ± 438.30 pg/mL and 429.39 ± 407.93 pg/mL, respectively (*p* < .001*vs.* baseline). Compared with baseline values (2.56 ± 0.19 mmol/L), serum calcium in this group declined to 2.11 ± 0.31 mmol/L at 20 min and 2.08 ± 0.19 mmol/L at 24 h after MWA, respectively (*p* < .0001 *vs*. baseline). Serum levels of calcium decreased to 2.10 ± 0.22 mmol/L on discharge days (*p* < .0001*vs.* baseline) but did not differ significantly from baseline at the end of follow-up (2.28 ± 0.52 mmol/L, *p* = .075 *vs.* baseline).

Before MWA, the serum iPTH in PHPT patients was 297.73 ± 295.32 pg/mL, and this decreased to 40.46 ± 26.29 pg/mL at 20 min after MWA (*p* = .002 vs. baseline) and 41.17 ± 32.93 pg/mL by the time of discharge (*p* = .003 *vs.* baseline) and 72.22 ± 36.51 pg/mL at the end of follow-up (*p* = .006 *vs.* baseline). The serum calcium level of the PHPT group was 2.72 ± 0.30 mmol/L before MWA, 2.43 ± 0.34 mmol/L at 20 min after MWA (*p* = .015 *vs.* baseline) and 2.46 ± 0.16 mmol/L on their discharge days (*p* = .004 *vs.* baseline) and 2.39 ± 0.11 mmol/L at the end of follow-up (*p* = .001 *vs.* baseline).

The target serum iPTH levels are in the range of 2–9 times the upper normal limit for CKD-5D patients [17]. After MWA treatment, nine SHPT patients had achieved the target range; four failed to reach the target; the other three died by the end of follow-up. One died of cardiopulmonary arrest during an amputation surgery due to infection with digital gangrene caused by calciphylaxis 9 months after ablation. Previous ablation therapy had not alleviated his finger pain. The causes of death of the other two patients were unknown. Therefore, the ratio of SHPT patients meeting the target was 69.23% (9/13). It was noteworthy that patients in target range still took medications to control serum iPTH or calcium levels after MWA. A male patient with serum iPTH of 56 pg/mL had stopped taking drugs by the end of follow-up.

#### Clinical symptoms and signs in SHPT patients before and after MWA

Most patients with PHPT were asymptomatic, and their elevated serum calcium and iPTH levels were revealed by routine health screening. However, renal SHPT patients manifested with bone pain, arthralgia, pruritus, ectopic calcifications in soft tissues, and rare calciphylaxis (Table 1). By the end of follow-up, the above symptoms had been alleviated to varying degrees (Table 5).

**Table 5.** Comparisons between pre-ablation and post-ablation symptoms in SHPT patients.

Symptoms/signs	Pre-ablation (*n* = 16)	Post-ablation (*n* = 13)*		
		No improvement	Improvement	Disappearance
Ostealgia	9	1 (11.1%)	7 (77.8%)	0
Arthralgia	12	2 (16.7%)	8 (66.7%)	0
Pruritus	5	1 (20.0%)	2 (40.0%)	0
Soft tissue ectopic calcification	2	1 (50.0%)	1 (50.0%)	/
Calciphylaxis	1	1	/	/

*Three patients died during the follow-up.

## Discussion

As an important aspect of CKD-MBD, SHPT affects the long-term survival and quality of life of patients. In recent years, parathyroid thermal ablations have been introduced as alternative treatments for PTX in severely affected SHPT patients with the risks of general anesthesia or older age [6,8]. PHPT is a common disorder that arises from autonomous overproduction of PTH by abnormal parathyroid glands [18]. Currently, most PHPT patients are asymptomatic at the time when they receive a diagnosis from routine laboratory testing results [19]. Different thermal ablation modalities have been employed in PHPT patients with promising results [20–22].

According to the Cardiovascular and Interventional Radiological Society of Europe (CIRSE) guidelines, ablative modalities are divided into chemical ablation and energy-based ablation. The latter includes radiofrequency ablation (RFA), microwave ablation, laser ablation, high-intensity focused ultrasound (HIFU), and cryoablation [13]. In patients with SHPT and PHPT, parathyroid ablations aim to ablate target glands by heating them to cytotoxic temperatures. To achieve cellular coagulative necrosis, temperatures must exceed 50 °C to 60 °C [23]. MWA works by using electromagnetic energy to force polar molecules (primarily H_2_O) in tissue to continuously realign with the oscillating electric field, hence increasing the temperature of the tissue [24]. Compared with RFA, MWA has the merits of shorter duration, higher temperatures, less sensitivity to tissue type, resistance to the heat-sink effect, and larger ablation volumes. Moreover, MWA is not influenced by tumor complexity [25].MWA may be more suitable for the ablation of parathyroid nodules than RFA [20].

Patients with severe SHPT who fail to respond to medication usually require PTX. In our study, the majority of SHPT patients were not eligible for PTX because of chronic heart failure with very low left ventricular ejection fraction, pulmonary hypertension, lung dysfunctions, etc. Only one SHPT patient received MWA treatment in accordance with his own will. On the other hand, PHPT patients had more freedom to choose the treatment approach because of their less comorbidities and young ages. MWA is an alternative therapeutic option for patients who either refuse surgery or are unsuitable candidates for surgery. It is the less invasiveness that makes it acceptable for the elderly who are at risk from general anesthesia and the young females who are concerned about the potential for scarring on their necks [26].

Hyperplastic parathyroid is easily detectable in the PHPT, while in SHPT patients more glands can be affected and each may have different degrees of hyperplasia or hypertrophy. Not always instrumental diagnostic investigations can detect all the parathyroid glands. Ectopic parathyroid glands can exist in various locations including the intrathyroid, carotid sheath, thymus, and upper mediastinum. If the targeted parathyroid glands are adjacent to important anatomical structures such as vessels or esophagus, they are unapproachable by percutaneous MWA either, thus causing persistent SHPT after MWA. Previous studies have shown that the frequency of ectopic parathyroid glands is about 15% [27], and the presence of supernumerary glands (more than four glands) ranges from 2.5% to 30% in SHPT patients [28]. Therefore, imaging of the parathyroid glands before MWA is important in SHPT patients to guarantee the safety and efficacy of treatment. The most commonly used diagnostic imaging techniques are USG and MIBI [29]. USG is recommended as a first modality because of its availability, non-invasiveness, and cost-effectiveness. USG can detect most parathyroid nodules, but it is occasionally difficult to differentiate an ectopic parathyroid nodule from an enlarged lymph node [20]. In the study, notably, the number of detected glands in a patient might vary according to different imaging modalities (Table 3). ^99m^Tc-MIBI SPECT/CT is the latest radionuclide imaging technology that provides the perfect combination of functional and anatomical information. Our previous study of 359 SHPT patients found that MIBI combined with SPECT/CT could increase the sensitivity and consistency of preoperative localization of eutopic parathyroid glands, and that it can accurately locate ectopic parathyroid [30]. Here, we found that the mean volume of each parathyroid gland in SHPT patients was relatively smaller than the PHPT group before MWA by ultrasound, although there was no significant difference. PHPT patients usually had only one enlarged hyperplastic parathyroid gland, whereas SHPT patients typically had multiple hyperplastic parathyroid glands due to their uremic environment. Furthermore, SHPT patients in the study were refractory to medical treatments and they have taken calcitriol or cinacalcet before MWA. The contrast-enhanced ultrasound (CEUS) is a promising technique to be used in the future, it based on the detection and characterization of microcirculation and microvascularization emerges as a reliable diagnostic tool to localize hyperplastic parathyroid glands [31]. Moreover, it is of utility in differentiating parathyroid adenomas from lymph nodes [32].

Coronary heart disease and atrial fibrillation are often encountered in patients with severe SHPT and frequently require antiplatelet or anticoagulant therapies. Up until now, there have been no hemorrhage risk scores for the prediction of bleeding events in uremic patients undergoing dual antiplatelet therapy (DAPT) with previous percutaneous coronary intervention (PCI). According to the European Society of Cardiology (ESC) guidelines, clopidogrel was recommended to be stopped for 5 days and prasugrel was recommended to be stopped for 7 days before surgery, with aspirin continued in surgical procedures with a high risk of bleeding. DAPT should be resumed as soon as possible within the first 24 h after operation [33]. In the present study, two severely affected SHPT patients were receiving DAPT after coronary stent implantations due to acute myocardial infarctions (AMI) within one year prior to MWA. Both patients were switched from aspirin and clopidogrel one week before MWA to low-molecular-weight heparin (LMWH) at 1 mg/kg q 12 h as periprocedural bridging therapy with heparin-free hemodialysis. LMWH was terminated on the ablation day, and DAPT was resumed on the next day.

The main complications encountered in the present study included recurrent laryngeal nerve injury and hypocalcemia. A ‘liquid insulation layer’ method or hydrodissection technique was adopted to protect the nerves. Additionally, the operator ablated the parathyroid glands at separate times to avoid bilateral recurrent laryngeal nerve injuries. Communication with the patient during MWA helped to detect any voice changes to prevent further damage. For patients who already have irreversible unilateral recurrent laryngeal nerve injury before MWA, clinicians should make them fully aware of the possibility of contralateral nerve injury. In the study, two patients had temporary hoarseness without dyspnea after ablation and they were treated with neurotropic agents such as mecobalamine and dexamethasone and nebulized inhalation therapies. Another remained slightly hoarse at the end of follow up. Transient hoarseness during MWA was encountered in three PHPT patients, all of whom recovered within 1 day after administration of nebulized and intravenous dexamethasone. The incidence of post-ablation hypocalcemia in SHPT patients was higher than in PHPT patients. This was probably because there were only one or two proliferative parathyroid glands in a PHPT patient with other glands functioning normally, while all of the enlarged glands in a SHPT patient needed ablating. Blood calcium levels should be monitored every 4–6 h within 24 h after ablation. The oral calcium supplement was given when the serum calcium <2.1 mmol/L. If blood calcium levels were below 1.8 mmol/L, intravenous pumping of calcium should be administered at an initial speed of 20 mL/h (adjusted on the basis of blood calcium levels) with 5% glucose solution (130 mL) added to 10% calcium gluconate (120 mL) to maintain the levels between 1.8 and 2.2 mmol/L.

In our research, 69.23% of SHPT patients achieved the serum target of iPTH after MWA according to the KDIGO guidelines. Compared with pre-ablation levels, serum calcium levels in SHPT patients decreased significantly on discharge days; then they began to rise into the normal range at the end of follow-up. Twelve SHPT patients still required medications by the end of follow-up. Symptoms in these patients, such as ostealgia, arthralgia and pruritus, had been alleviated by the end of follow up. The serum iPTH levels of PHPT patients decreased significantly at the end of follow-up compared with pre-ablation levels and were within the normal range. A continuing downward trend in serum calcium levels could be observed in this group. None of the PHPT patients required medications after MWA.

Possible reasons for the rebound of serum iPTH levels after MWA in SHPT patients include the following. First, percutaneous ultrasound-guided MWA is different from PTX, which provides a clear surgical field in which all parathyroid glands can be removed. Second, some parathyroid glands were relatively deep or close to important vessels and nerves. To ensure the safety of MWA, the operator had to weigh the pros and cons in the individual patient to decide whether to ablate all of the glands. Third, there might be some ectopic or immature proliferative parathyroid glands, detecting these glands on imaging examinations may be difficult. Under the continuous stimulation of uremic environmental disorders, residual parathyroid cells or glands can proliferate, leading to a recurrence of SHPT after ablation. SHPT patients were counseled to continue medical therapy after MWA, including active vitamin D analogs or cinacalcet. If necessary, another ablation may be appointed.

With the merits of minimal invasiveness, local anesthesia, more rapid postoperative recovery, and repeated operations, MWA is suggested for SHPT and PHPT patients who are ineligible or reluctant for PTX. However, the preoperative, intraoperative and postoperative treatments of the two diseases were distinguished. Compared to PHPT patients, SHPT patients had more prominent malnutrition, cardiovascular/pulmonary diseases, and abnormal bone metabolism, which require a more careful and comprehensive workup before MWA. PHPT patients had larger volumes and maximum diameters of single glands and longer ablation times for each gland. SHPT patients usually underwent MWA treatments at two separate times in order to avoid bilateral recurrent laryngeal nerve injuries, whereas PHPT patients were treated only once. SHPT patients were more likely to develop hypocalcemia, and their serum iPTH levels may rebound after MWA. Therefore, all of the hyperplasic parathyroid glands found on ultrasound should be ablated completely in SHPT patients. After MWA, patients are suggested to be followed up regularly and treated pharmacologically if necessary, especially in SHPT group (Table 6).

**Table 6.** Differences between MWA in PHPT and SHPT patients.

	PHPT	SHPT
Comorbidities	Less common	Common
Perioperative evaluation	Routine	More comprehensive
Procedure duration	Longer time for ablating a single gland	Multiple ablations in separated times
Efficacy	Satisfactory	Satisfactory
Post-ablation horseness	Transient	Transient, permanent on occasion
Posta-ablation hypocalcemia	Less common	Common, needs correcting timely
Follow up	Regularly	Regularly

There were several limitations in this study. First, this was a retrospective single-center study with a small sample. Second, a longer follow-up duration and more follow-up indexes are needed to determine long-term efficacy comprehensively. Third, the majority of SHPT patients still took calcitriol or cinacalcet to control their diseases after ablation. Until now, PTX is still the first choice of treatment for severely affected SHPT patients. How to improve the efficacy of MWA in the treatment of SHPT patients who are ineligible for PTX remains to be solved.

## Supplementary Material

Supplemental Material

Supplemental Material

Supplemental Material

Supplemental Material
